# Rapid Granulation Tissue Regeneration by Intracellular ATP Delivery-A Comparison with Regranex

**DOI:** 10.1371/journal.pone.0091787

**Published:** 2014-03-17

**Authors:** Jeffrey D. Howard, Harshini Sarojini, Rong Wan, Sufan Chien

**Affiliations:** Department of Surgery, University of Louisville, Louisville, Kentucky, United States of America; Institute for Frontier Medical Sciences, Kyoto University, Japan

## Abstract

This study tests a new intracellular ATP delivery technique for tissue regeneration and compares its efficacy with that of Regranex. Twenty-seven adult New Zealand white rabbits each underwent minimally invasive surgery to render one ear ischemic. Eight wounds were then created: four on the ischemic and four on the normal ear. Two wounds on one side of each ear were treated with Mg-ATP encapsulated lipid vesicles (ATP-vesicles) while the two wounds on the other side were treated with Regranex. Wound healing time was shorter when ATP-vesicles were used. The most striking finding was that new tissue growth started to appear in less than 1 day when ATP-vesicles were used. The growth continued and covered the wound area within a few days, without the formation of a provisional matrix. Regranex-treated wounds did not have this growth pattern. In wounds treated by ATP-vesicles, histologic studies revealed extremely rich macrophage accumulation, along with active proliferating cell nuclear antigen (PCNA) and positive BrdU staining, indicating in situ macrophage proliferation. Human macrophage culture suggested direct collagen production. These results support an entirely new healing process, which seems to have combined the conventional hemostasis, inflammation, and proliferation phases into a single one, thereby eliminating the lag time usually seen during healing process.

## Introduction

Chronic wounds affect 6.5 million patients in this country, with a treatment cost of more than $25 billion each year [1], [2]. One form of chronic wounds, the diabetic foot ulcer, develops in fifteen to twenty-five percent of diabetic patients in their lifetimes [3], [4]. Despite the development of thousands of dressings, the best treatments currently available achieve only a 50% healing rate and even this is often temporary [5], as shown by high recurrence rates (66% for diabetic ulcers) [1], [2]. At present, the only supplement that has successfully completed randomized clinical trials in the USA is the recombinant human platelet-derived growth factor-BB (rhPDGF-BB, Regranex); it has been approved by the FDA for treatment of diabetic neuropathic ulcers [6] and has also been approved by American and European authorities for healing of other chronic wounds [7], [8]. Regranex has been reported to enhance cell migration and granulation tissue growth—two critical factors in chronic wound healing. On the other hand, disappointing results have also been reported, both clinically and experimentally [9]–[11], but no other dressing to date has shown better results than Regranex.

We previously reported a technique in which adenosine triphosphate (Mg-ATP) was encapsulated within very small unilamellar lipid vesicles (ATP-vesicles). We used full-thickness skin wounds in a rodent model to test these vesicles. The healing was enhanced, but skin contraction probably contributed to the healing process [12]. However, skin contraction contributes little to overall human chronic wound healing, so translation of these early animal results to human chronic wounds is difficult. We also used this preparation to treat full-thickness skin wounds in rabbits and obtained very good results in the form of fast granulation tissue growth [12]–[14]. The results were believed to reflect the increase in energy supply, which caused early stem cell and leukocyte trafficking, accumulation, and differentiation [15]. In the present study, we examined how wound treatment with ATP-vesicles compared with Regranex in terms of cell activity, tissue growth, and wound healing in a model devoid of skin contraction, with and without ischemia. We hypothesized that the energy supplied by the intracellular delivery technique would facilitate the healing process in a different way from the traditional healing process achieved with Regranex. The results obtained from these experiments have provided support for this hypothesis.

## Materials and Methods

### Preparation of ATP-vesicles

The ATP-encapsulated unilamellar lipid vesicles (ATP-vesicles) were produced by Avanti Polar Lipids Inc. (Alabaster, AL) and provided to us in a freeze-dried form. They were stored at −20°C and were reconstituted with normal saline immediately before use. After reconstitution, the composition was: 100 mg/ml of Soy PC/DOTAP (50∶1), Trehalose/Soy PC (2∶1), 10 mM KH_2_PO_4_ and 10 mM Mg-ATP. The diameters of the lipid vesicles ranged from 120–160 nm, as measured with a DynaPro Particle Size Analyzer (Proterion Corporation, NJ).

### Animals and Wounds

This study was conducted in accordance with the National Institutes of Health guidelines for the care and use of animals in research, and the protocol was approved by the Institution Animal Care and Use Committee of the University of Louisville, an AAALAC accredited program. Twenty-seven adult New Zealand white rabbits (2.0–3.0 kg, Myrtle's Rabbitry, Thompson Station, TN; and Harlan Laboratories, Indianapolis, IN) were used: 9 rabbits (72 wounds) were used for healing time and granulation tissue growth comparison, and the remaining 18 rabbits were sacrificed from 5 hours to 27 days post-operation for histologic and immunohistochemistry studies.

We created ischemic wounds using a minimally invasive technique previously developed and further simplified in our laboratory [16], [17]. This technique creates one ischemic ear while the other ear is used as a non-ischemic control. Briefly, under general anesthesia (Ketamine 50 mg/kg and Xylazine 5 mg/kg, IM), three small incisions were made on the ear base. The central artery and cranial vessels, along with their accompanying nerves, were severed, leaving only the central vein and caudal vessels intact. The skin incisions were closed. The other ear was preserved as a non-ischemic control. Four full-thickness skin wounds (6 mm in diameter) were made to the depth of the cartilage on the ventral side of each ear with a stainless steel punch. The perichondrium was removed with the skin or separately. The base of the wound consisted of cartilage but the cartilage itself was not perforated.

### Postoperative Management

Postoperatively, all animals received analgesics in the form of a fentanyl patch (25 μg/hour) and buprenorphine (0.01 mg/kg, IM) for 2–3 days to reduce possible pain. The animals were allowed free access to food and drink. The ischemic ears were observed daily for blood circulation and the closed incisions were examined for signs of bleeding or infection. Ear skin temperature was measured and recorded daily. The wounds were treated with two types of dressings: The two wounds on one side of the ear were treated with ATP-vesicles (10 mM ATP), while the control wounds on the other side of the same ear received Regranex (100 μg/g). A small piece of sterile paper was used to cover the dressings and the wound was further covered with TegaDerm (3 M, Minneapolis, MN) to prevent desiccation. Dressings were changed daily until healing or sacrifice. The old dressings were removed, the wounds cleaned, digital photos taken with a scale, and new dressings were applied.

### Wound area, Cavity, and Reepithelization Measurements

Three different techniques were used to analyze the wound healing process: 1) Wound closure time. Wound size reduction and reepithelialization were monitored daily until total reepithelialization occurred and covered all exposed granulation tissue; 2) Wound cavity reduction. This was measured by tracing the granulation growth and the cavity edge on two-dimensional digital images obtained daily to determine the wound cavity area (not volume because the image is two-dimensional); and 3) Wound reepithelialization rate. This was measured daily by tracing the epithelialized areas from the wound edge towards center, calculating the remaining non-epithelized area, and comparing it to the original area. This tracing may not be precise in the wounds treated with ATP-vesicles due to overgrowth of granulation tissue, which often overtook the wound edge while the reepithelialization tunneled though the granulation tissue [13]. These measurements were performed morphometrically using digital images of the wounds obtained daily with scales and processed with the NIH image software ImageJ, which allows precise tracing of irregular lines and area calculations, similar to other software used as a standardized method in the past [18], [19].

### Histology and Immunohistochemical Studies

Immediately post-euthanasia, a circular section of a sample that included the whole wound and a ring of its surrounding tissue (approximately 2–3 mm in width), was excised using a steel punch, immersed in 10% buffered formalin, processed with the Thermo-Fisher Pathcentre (Fisher Scientific, Chicago, IL), embedded in paraffin blocks, and cut into 5 μm sections. One set was stained with hematoxylin and eosin (H & E) for general histologic evaluation. Collagen deposition in the wound area was assessed by van Gieson (HT254, Sigma-Aldrich, St. Louis, MO) and picrosirius red (Polysciences, Warrington, PA) staining [20]. Macrophage infiltration in the wound tissue was detected by immunohistochemical staining using anti-MAC387 antibody (AbD Serotec, Raleigh, NC) and further confirmed with CD68 (Dako, Carpinteria, CA) and CD163 (Abcam, Cambridge, MA) antibodies [21]. Cell proliferation was determined with proliferating cell nuclear antigen (PCNA) antibodies (Santa Cruz Biotech, Dallas, TX) and further confirmed by BrdU (Sigma, St. Louis, MO). For BrdU labeling, the reagent was injected peritoneally (10 ml concentrated reagent/kg body weight) 24 hours before sacrifice and the paraffin-fixed samples were stained with anti-BrdU antibody (Millipore, Billerica, MA).

Histology images were analyzed with a Nikon Eclipse Ti microscope (Nikon Instruments Inc.) and collagen types of picrosirius red stained slides were further analyzed with a Zeiss AxioScope circular polarizing microscope (Carl-Zeiss, Thornwood, NY). The images captured by the computer were further quantified morphometrically using Nikon Elements or Zeiss Axio Imaging software.

### Macrophage Culture and Measurements

We further explored macrophage proliferation, transformation, and collagen production in a more exclusive environment by performing a primary culture study using human macrophages. The protocol was approved by the University of Louisville Institutional Review Board, and signed consent was obtained from each participant. A total of 16 healthy volunteer donors, age 19–49 years, were enrolled; most of the experiments were conducted with five donors per group. Venous blood was collected in EDTA Vacutainers (Becton Dickinson, Franklin Lakes, NJ). Monocytes/macrophages were isolated using the magnetic cell sorting technique according to manufacturer's instructions (Miltenyi Biotec, Auburn, CA). The purity of the isolated monocytes/macrophages was >95% as determined by flow cytometry. The primary monocytes were cultured in 1640 RPMI medium (MP Biomedicals, Solon, OH) supplemented with 10% heat-inactivated defined fetal bovine serum, 2 nM L-glutamine, 100 IU/ml penicillin, 100 μg/ml streptomycin, and 250 ng/ml amphotericin B (Thermo Scientific, Waltham, MA). Cells were plated in 24 well culture plates at 0.5×10^6^ cells/ml/well and incubated in a humidified incubator with 5% CO_2_ at 37°C. Twenty-four hours post plating, the attached cells were treated with ATP-vesicles (0.1, 1, and 10 mM Mg-ATP), 1 mM free Mg-ATP, 1 mM lipid vesicles (without Mg-ATP), 0.001% Regranex, or culture medium alone for an additional 24 hrs. Collagen type 1α1 production was determined by ELISA.

### Enzyme-Linked Immunosorbent Assays (ELISA)

Collagen type 1α1 levels were determined from the culture supernatant by enzyme-linked immunosorbent assays (My Biosource LLC, San Diego, CA) in 96-well plates according to the manufacturer's protocol. All samples were analyzed in triplicate; collagen levels of samples were determined based on a standard curve using standard collagen provided in the kit.

### Measurement of Cell Viability

Cell viability was determined by the 3-[4,5-dimethylthiazol-2-yl]-2,5-diphenyltetrazolium bromide (MTT) assay. Primary monocytes/macrophages (1×10^6^ cells) were seeded into 96-well microtiter plates with 100 μL RPMI media. Twenty-four hours post plating, the attached cells were treated with ATP-vesicles (1 mM ATP), Regranex (10 μm/g), or lipopolysaccharides (LPS, 100 ng) for an additional 5 days. After the incubation period, the media were removed from each well and 100 μL MTT solution (2 mg/mL; Sigma-Aldrich, MO) and phosphate-buffered saline (PBS) were added to each well. Plates were incubated at 37°C for 4 h. The MTT solution was then removed, leaving purple formazan crystals that were subsequently dissolved in 100 μL dimethylsulfoxide. The absorbance of each well was then read at 540 nm using a Plus384 spectrophotometer (Molecular Devices, CA).

### Statistical Analysis

Results are reported as means and standard deviation (SD). The ATP-vesicles and Regranex-treated wounds or cells were compared by the Student t-test using a commercially available spreadsheet (Microsoft Excel) and statistical software including GraphPad Prism (GraphPad Software, La Jolla, CA), SPSS (IBM), and JMP 10 (SAS Campus Drive, Cary, NC). A *p* value of <0.05 was considered significant.

## Results

### General Appearance

**Figure 1:**
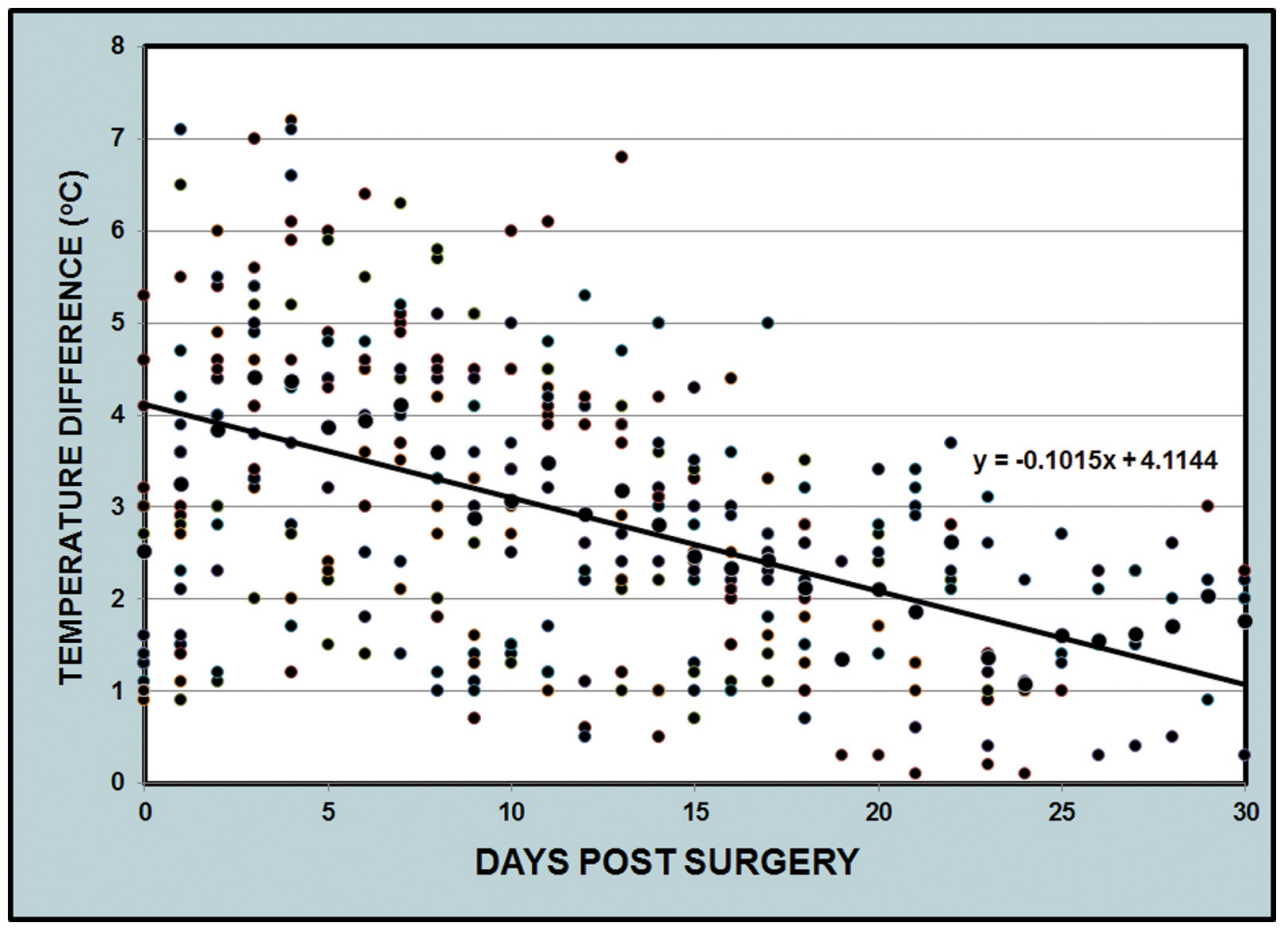
Skin temperature differences between the non-ischemic ears and ischemic ears. The difference was larger at the beginning of the experiment, but decreased gradually over time. However, a temperature difference of more than 1°C was maintained at the end of one month.

In the animal experiments, the postoperative ischemic ear became cool and mild cyanotic, with a reduced sensation distal to the incision. The mean skin temperature differences between the normal (non-ischemic) ears and ischemic ears ranged from 0.9 to 7.1°C (mean 4.2°C) immediately after surgery. This temperature decreased gradually over time but was still maintained above 1°C at the end of one month (Figure 1). The most important ear artery, the central artery, had a strong pulse in the normal ear, but this pulse was absent in the ischemic ear. The ischemic ear movement was reduced but not totally eliminated because some muscles were still attached to the base of the ear.

### Wound Measurements

**Figure 2:**
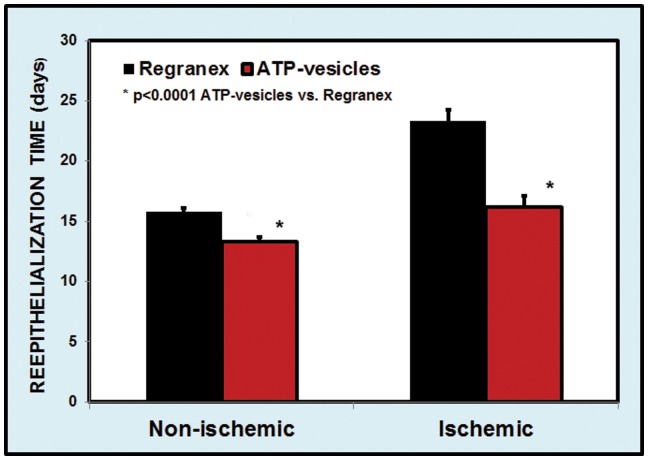
Comparison of complete reepithelialization times between wounds treated with ATP-vesicles and those treated with Regranex.

**Figure 3:**
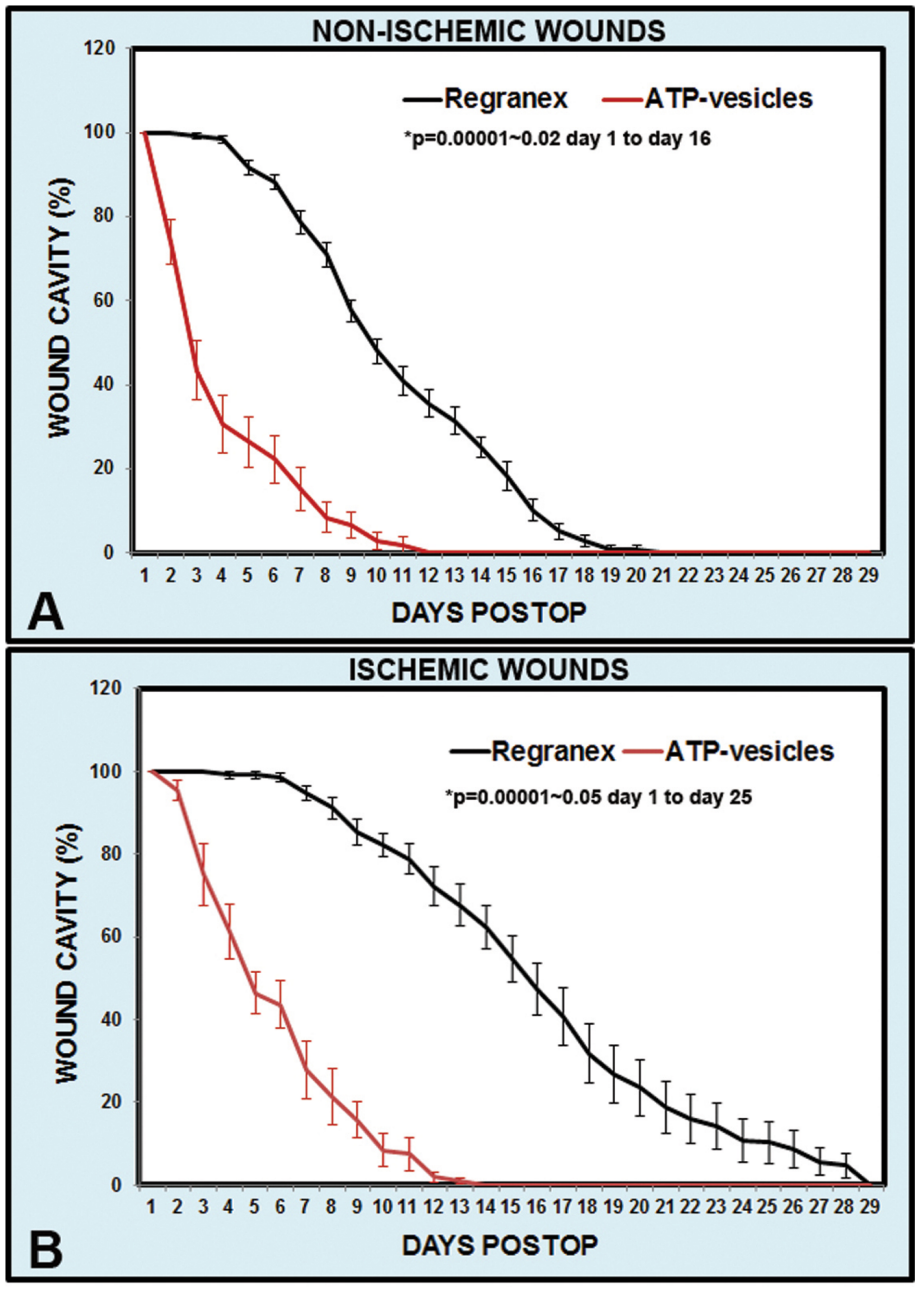
Comparison of wound cavity size reduction rate in wounds treated with ATP-vesicles and those treated with Regranex. (A) Non-ischemic wounds; (B) Ischemic wounds.

The speed of healing and reepithelialization was enhanced by the ATP-vesicle treatment. On the non-ischemic ears, the average time for complete reepithelialization was 13.0 ± 0.4 days for ATP-vesicle-treated wounds versus 16.0 ± 0.3 days for Regranex-treated wounds (n = 18, p<0.0001). On the ischemic ear, the average total reepithelialization time was 16.2±0.9 days for the ATP-vesicle-treated wounds versus 23.3 ± 0.9 days for Regranex-treated wounds (n = 18, p<0.0001) (Figure 2). Sequential daily reepithelialization comparisons also indicated a faster reduction in non-reepithelialized areas in wounds treated with ATP-vesicles. Due to the very rapid granulation tissue regeneration in the wounds treated with ATP-vesicles, wound cavity reduction was much faster in these wounds: In non-ischemic wounds, a 50% reduction in the wound cavity area took an average of 3.0 days in wounds treated with ATP-vesicles, while it took an average of 10.3 days in Regranex-treated wounds. In ischemic wounds, a 50% reduction in the wound cavity took an average of 4.7 days for ATP-vesicle treated wounds while it took an average of 15.5 days for Regranex-treated wounds (Figure 3). The real wound cavity reduction was probably even more rapid in wounds treated with ATP-vesicles since, in many of these wounds, granulation tissues grew higher than the surrounding skin and the seeming defects were actually resting on top of the granulation tissue that had already filled the wound cavity.

### Extremely Rapid Tissue Regeneration

One unprecedented finding in this study was the very early and rapid generation of granulation tissue in ATP-vesicle treated wounds: new tissue growth started to appear within 24 hours after surgery and sometimes as early as 12 hours. The growth continued and covered the defects quickly. The early growth appeared to be somewhat pale and edematous, but it solidified and gradually changed to a pink color after 2–3 days. Reepithelialization tunneled through this seeming overgrowth and the top portion became a scab, which fell off to reveal perfectly healed defects without any hypertrophic scar formation. No provisional matrix was seen in these wounds.

**Figure 4:**
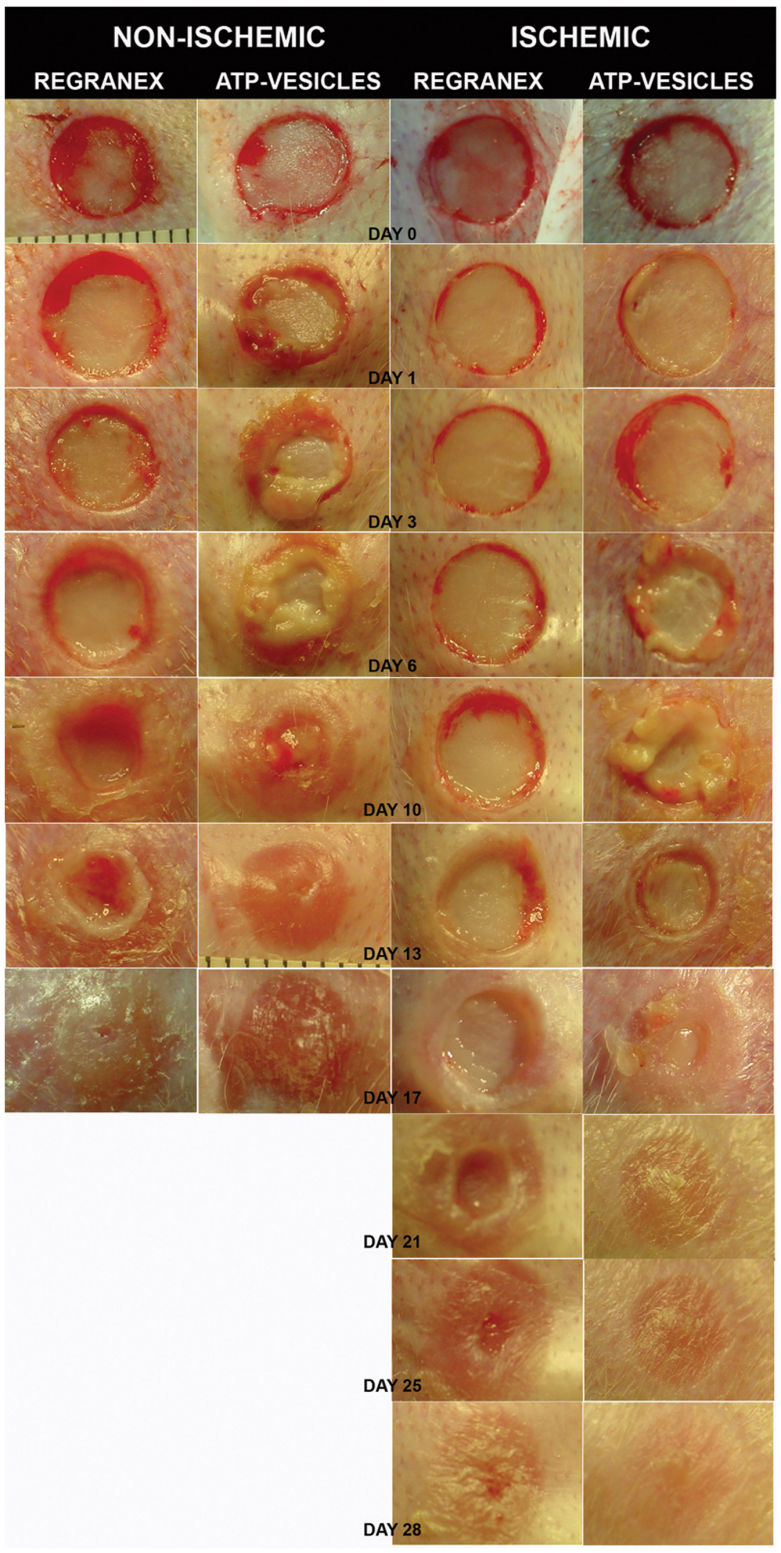
Granulation comparison. A representative comparison between the wounds treated with ATP-vesicles and Regranex. Granulation growth starts to occur one day after surgery in non-ischemic wounds when treated with ATP-vesicles. In ischemic wounds, the granulation occurs a few days later, but wounds treated with ATP-vesicles still heal faster than those treated with Regranex.

Regranex-treated wounds showed a completely different growth pattern in which no such granulation tissue was seen; these wounds healed gradually from the margin. One typical comparison is shown in Figure 4.

**Figure 5:**
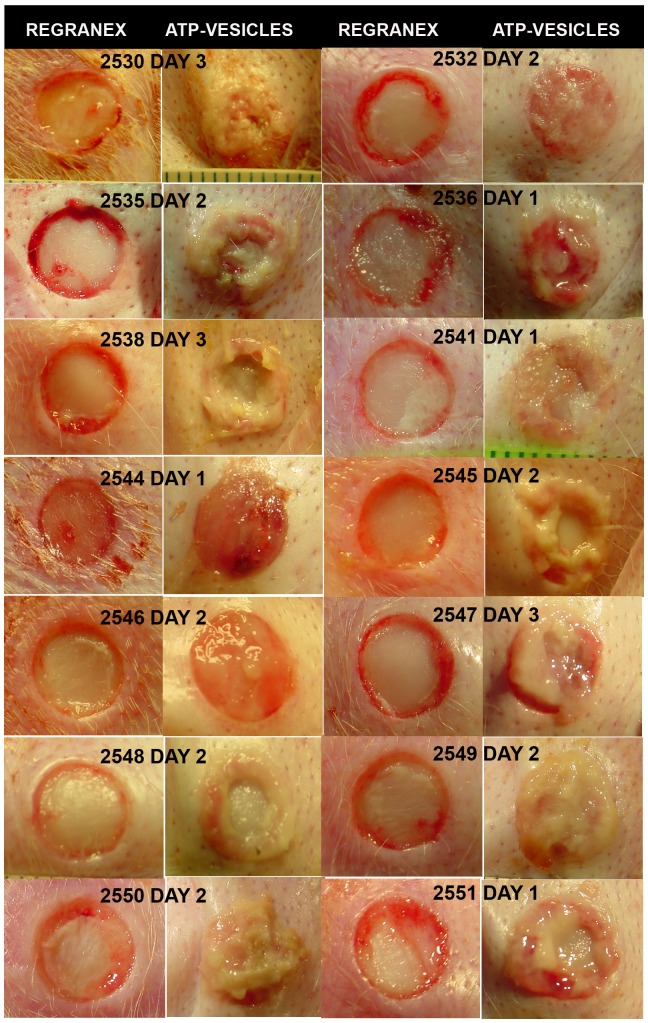
More comparisons of granulation tissue. All ATP-vesicle treated wounds start to generate granulation tissues within 1–3 days while the wounds treated with Regranex show no such growth.

Ischemic wounds showed granulation that occurred a few days later, but wounds treated with ATP-vesicles again healed faster than those treated with Regranex. Figure 5 shows more examples in which all ATP-vesicle treated wounds started to generate granulation tissues within 1–3 days; note that the wounds treated with Regranex showed no such growth.

### Microscopy Studies

**Figure 6:**
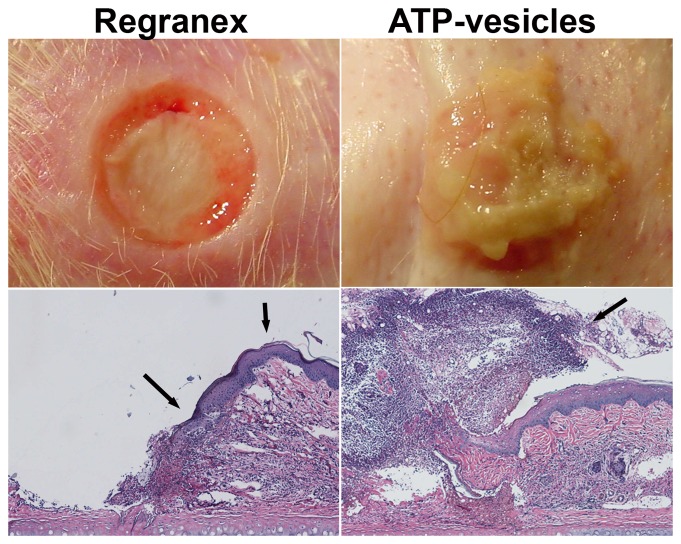
Representative photographs and H&E staining wounds at day 3 of treatment. The wound treated with ATP-vesicles produces new tissue growth that has completely covered the wound bed and overtaken the wound edge (arrow). The growth is filled with cells. The wound treated with Regranex produces a hypertrophic rim of tissue, which sometimes grows inward (40x, arrows).

Microscopy studies indicated significant differences between the ATP-vesicle group and the Regranex group. Massive cell accumulation occurred in the granulation tissues of wounds treated with ATP-vesicles, while the Regranex group showed no growth. Figure 6 shows a representative day 3 photographic comparison between the two groups. The ATP-vesicle-treated wound is covered by new tissue growth that has overtaken the wound edge. H & E staining indicates massive cell accumulation in the wound treated with ATP-vesicles. The wound treated with Regranex has no such growth, but instead shows a hypertrophic rim of tissue isolated to the wound border. Occasionally, the rim grew inward towards the wound bottom.

**Figure 7:**
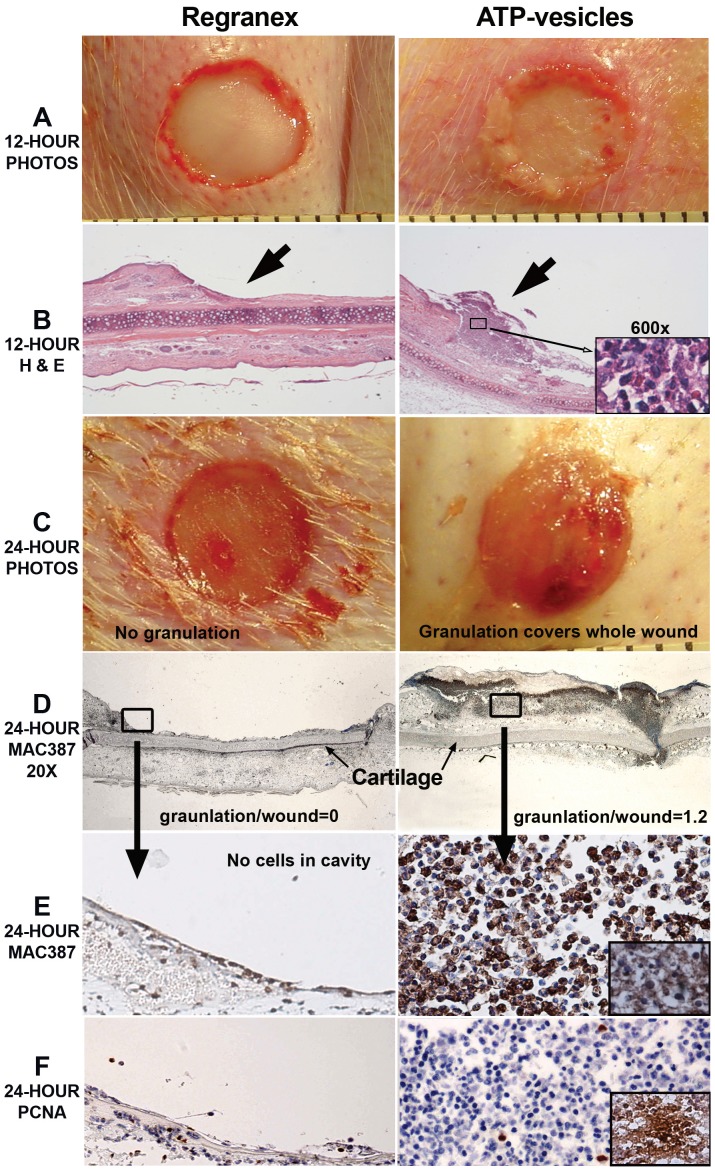
A photomontage of wounds and microscopic changes occurring within 24 hours after surgery. When ATP-vesicles are used, granulation starts to appear within 12 hours (A), and H&E staining indicates a rich cellular component (B). Granulation tissue growth continues and covers the whole wound at 24 hours (C). Granulation tissue shows positive anti-MAC387 staining (D), which is further confirmed by CD163 staining (inset) (E). PCNA staining indicates very active proliferation of these cells (F), which is further confirmed by BrdU antibody staining (inset). Wounds treated with Regranex do not display this rapid growth.

Figure 7 is a combination photomontage of gross photos, H&E stains, and immunohistochemistry stains taken from 12 to 24 hours after wounding. At 12 hours, gross granulation tissue growth is already seen in the ATP-vesicle treated wounds, while the wounds treated with Regranex have no such growth (Figure 7A). Microscopically, the growth promoted by ATP-vesicles is full of cells (Figure 7B). By 24 hours, this wound is already totally covered by granulation tissue—it is somewhat edematous rather than very solid (Figure 7C). However, IHC staining shows a solid cell mass mainly composed of macrophages, as shown by anti-MAC387 and further confirmed by CD68 and CD163 staining (Figure 7D & E). Very active cell proliferation is determined with PCNA, and further confirmed by BrdU antibody staining (Figure 7F).

### Macrophage Counts

**Figure 8:**
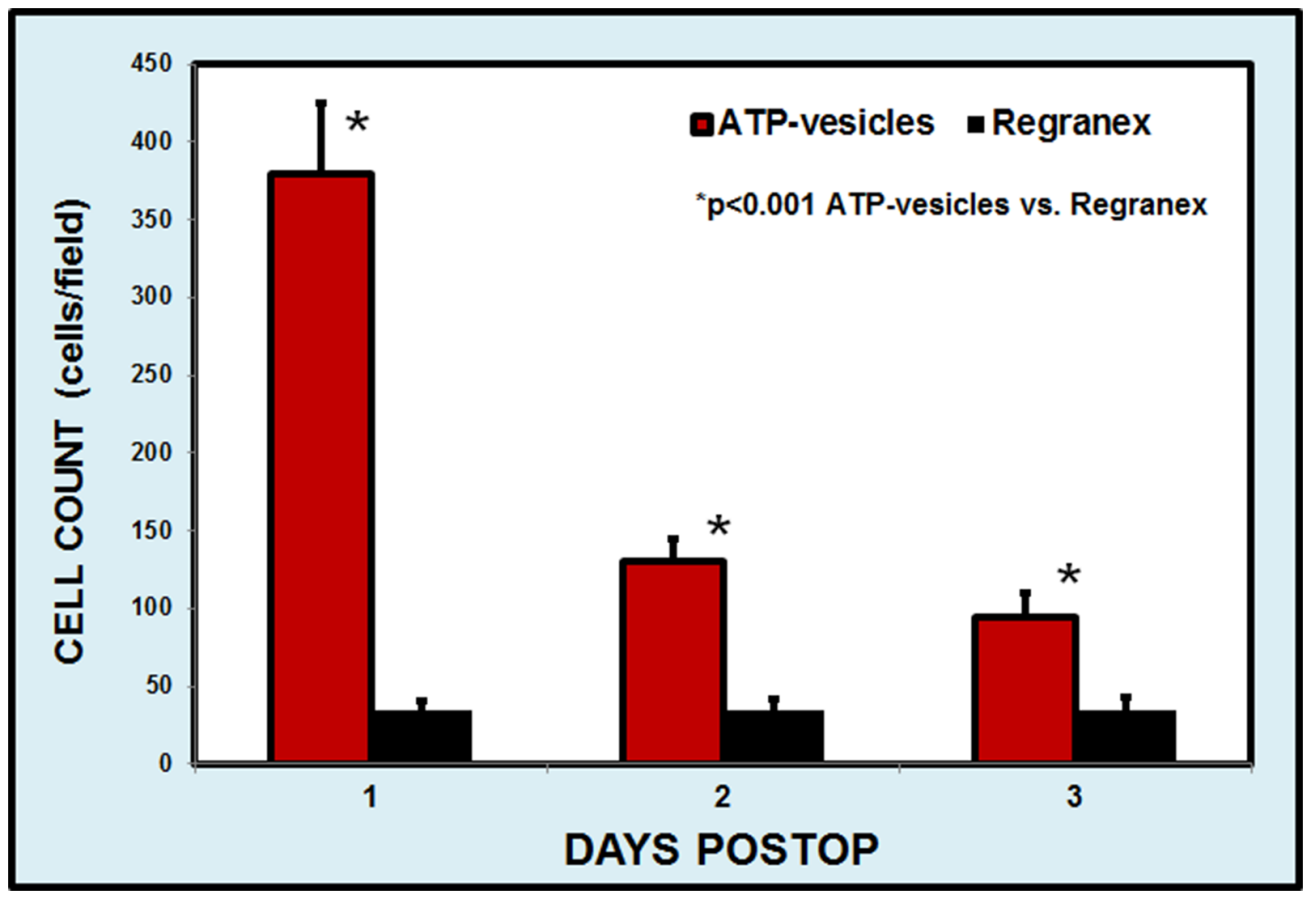
Comparison of macrophage numbers in the early days of the healing process. Regranex-treated wounds show a slight increase in macrophage numbers. However, when ATP-vesicles are used, macrophage numbers are 7–8 times higher than in the Regranex-treated wounds at day 1, but decrease quickly thereafter.

**Figure 9:**
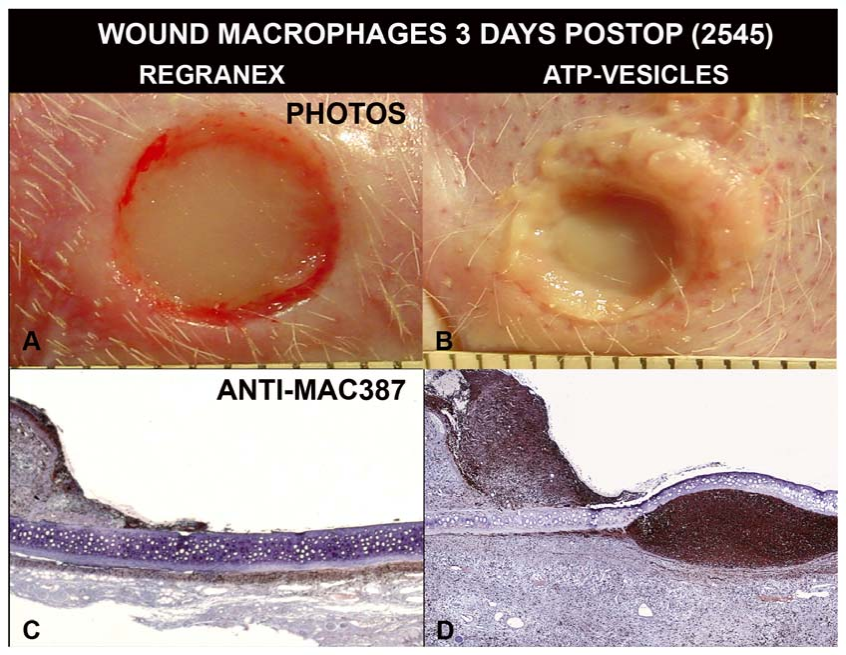
Macrophage accumulation in early days. When the wounds are treated with ATP-vesicles, solid granulation occurs at day 3 (B). The growth is filled with macrophages, which occur not only in wound cavity, but also underneath the ear cartilage (D). The wounds treated with Regranex do not have similar growth (A, C).

A careful macrophage number count in the wound area revealed a significant difference in macrophage numbers between the ATP-vesicle-treated and Regranex-treated wounds. Macrophage numbers in the Regranex-treated wounds showed only a slight increase similar to that seen in the traditional healing pattern [22], [23]. However, when ATP-vesicles were used, macrophage numbers increased sharply at day 1, but decreased quickly thereafter (Figure 8). This is significantly different from traditional macrophage behavior in wound tissue treated with any other dressings. On occasion, macrophage accumulation following ATP-vesicle treatment occurred well beyond the wound area, so that the accumulation was not only seen in the wound wall and cavity but also underneath the ear cartilage, indicating deep penetration of the ATP-vesicles (Figure. 9).

### Wound Collagen Study

**Figure 10:**
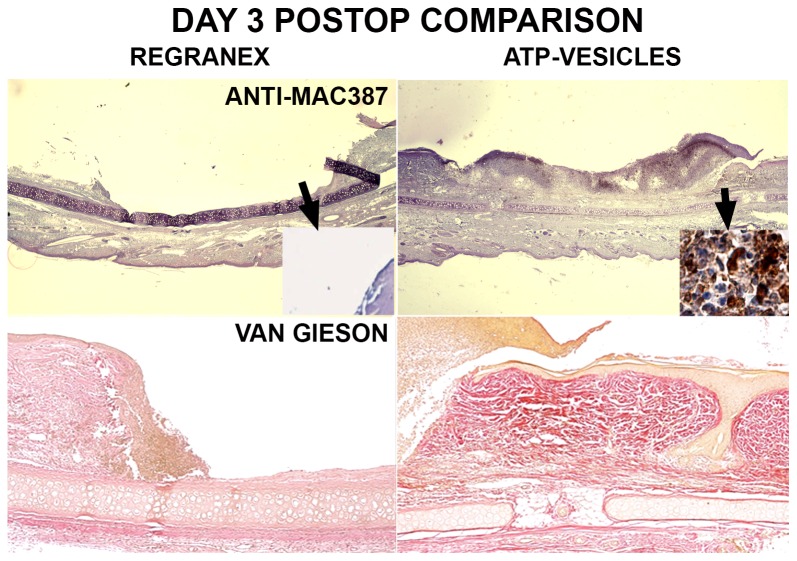
Comparison of Regranex and ATP-vesicle treated wounds 3 days postoperatively. Rich collections of macrophages are stained dark brown to black by Anti-Mac387 (top panels), and more collagen is shown by van Gieson stain (low panels) in the wounds treated with ATP-vesicles. Regranex treated wounds do not show a similar effect.

**Figure 11:**
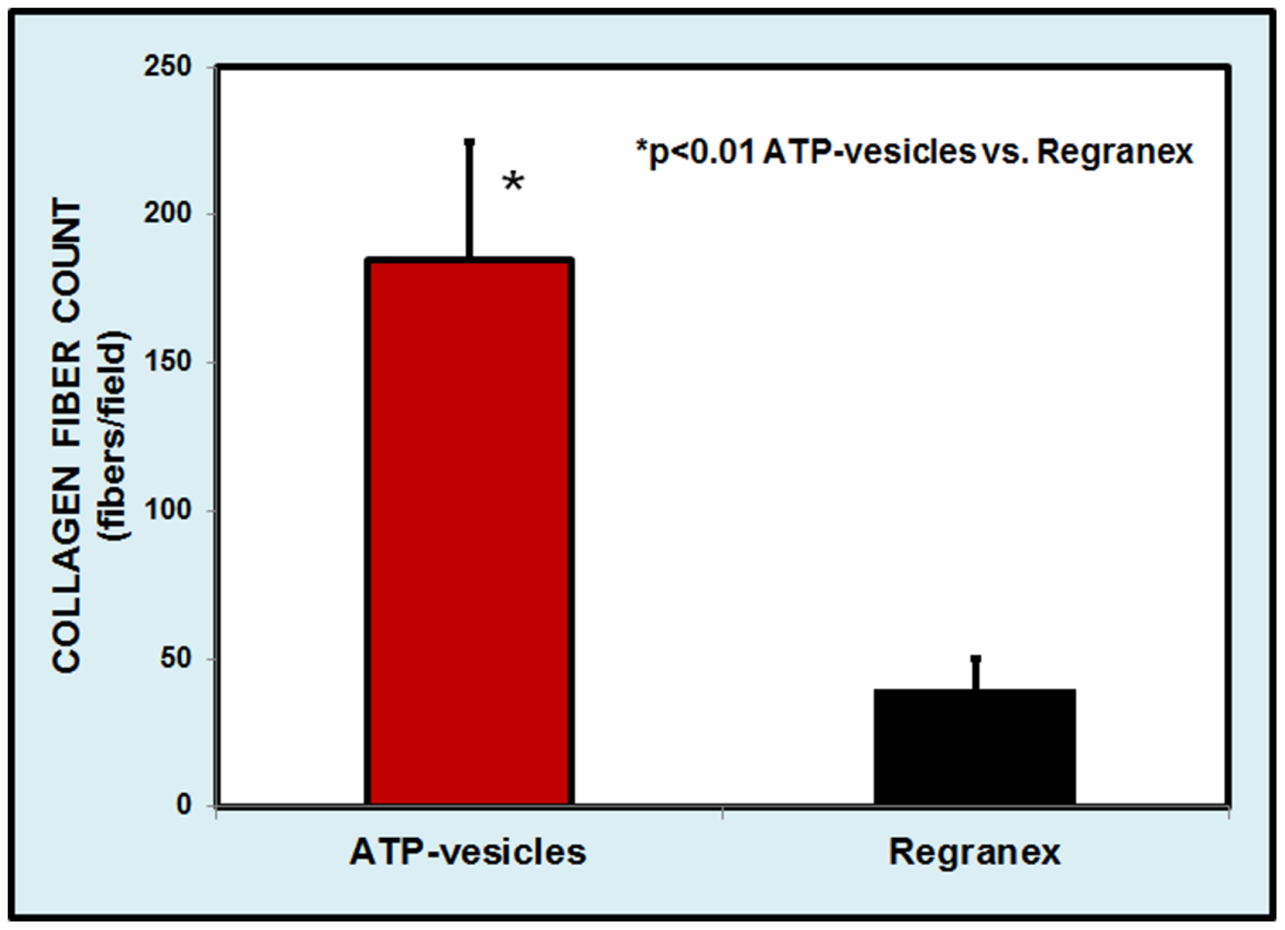
Comparison of direct collagen fiber counts 3 days after surgery. Wounds treated with ATP-vesicles show 4–5 times higher collagen content than those treated with Regranex.

Wound tissue collagen production was also correlated with macrophage proliferation and accumulation: when significant macrophage accumulation was noted, more collagen appeared in the wound tissue. Figure 10 shows a day 3 photo comparison. In the wound treated with ATP-vesicles, granulation tissue had already covered the whole wound and had overtaken the wound edge. At the same time, rich macrophage accumulation was indicated by Anti-Mac387 and higher collagen accumulation was shown by van Gieson staining. However, the wound treated with Regranex had no such growth or collagen accumulation. Collagen fiber count indicated much higher collagen content in the wounds treated with ATP-vesicles than in those treated with Regranex (Figure 11).

### Macrophage Survival and Collagen Production in Cell Culture

**Figure 12:**
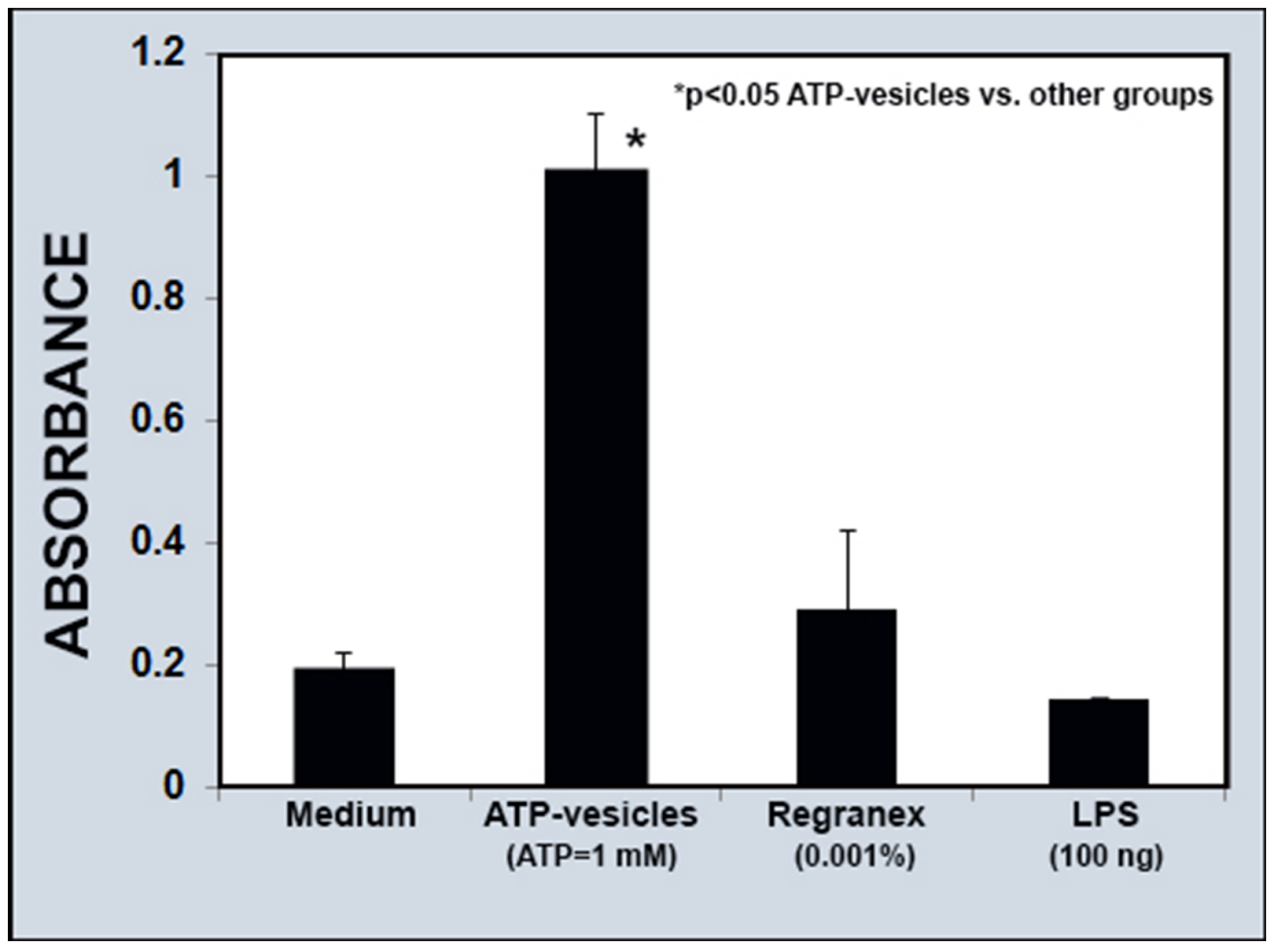
Comparison of cell survival in culture. The addition of ATP-vesicles (1 mM Mg-ATP) into the culture medium increases monocyte-macrophage survival time 3–4 folds compared to the culture medium alone, Regranex (0.001%), or LPS (100 ng).

The addition of ATP-vesicles to cell cultures seemed to significantly enhance monocyte-macrophage survival time. As seen in Figure 12, the use of ATP-vesicles (1 mM ATP) extended human monocyte/macrophage survival 3–4 fold when compared to cells treated with medium alone, Regranex (0.001%), or LPS (100 ng), as determined by the MTT assay.

**Figure 13:**
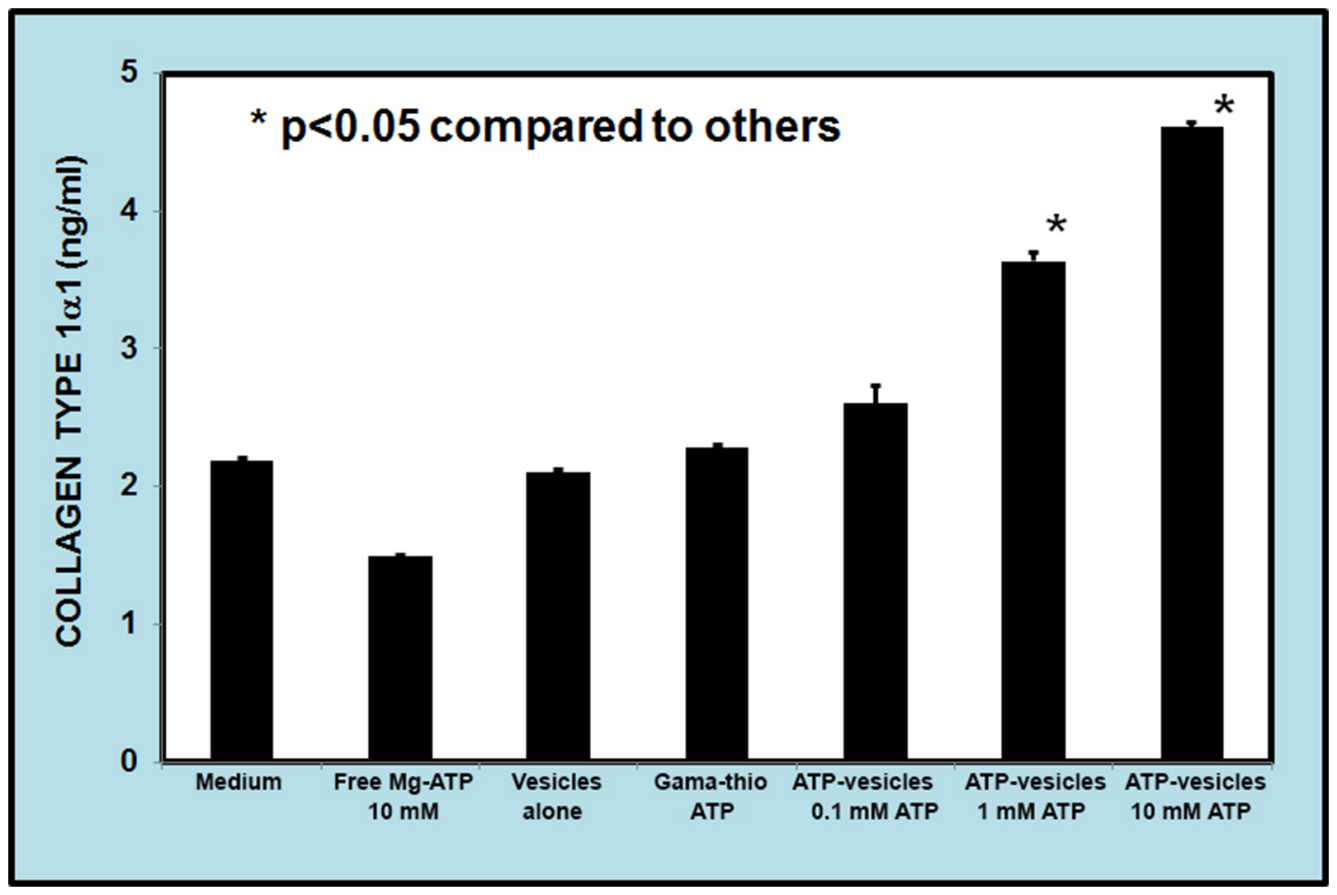
Dose-dependent collagen production by macrophages. Macrophage collagen production appears to be dose-dependent when ATP-vesicles are used. The higher the ATP concentration, the more collagen 1α1 is produced.

The ability of macrophages to produce collagen was also measured in macrophage cultures. Figure 13 shows collagen type 1α1 production in cell culture in response to addition of free Mg-ATP, free lipid vesicles, ATP-vesicles containing gamma-thio-ATP (a non-hydrolysable ATP), and lipid vesicles containing various concentrations of Mg-ATP. None of the vesicle components increased collagen production when supplied alone, but increasing concentrations of encapsulated ATP induced more collagen production.

**Figure 14:**
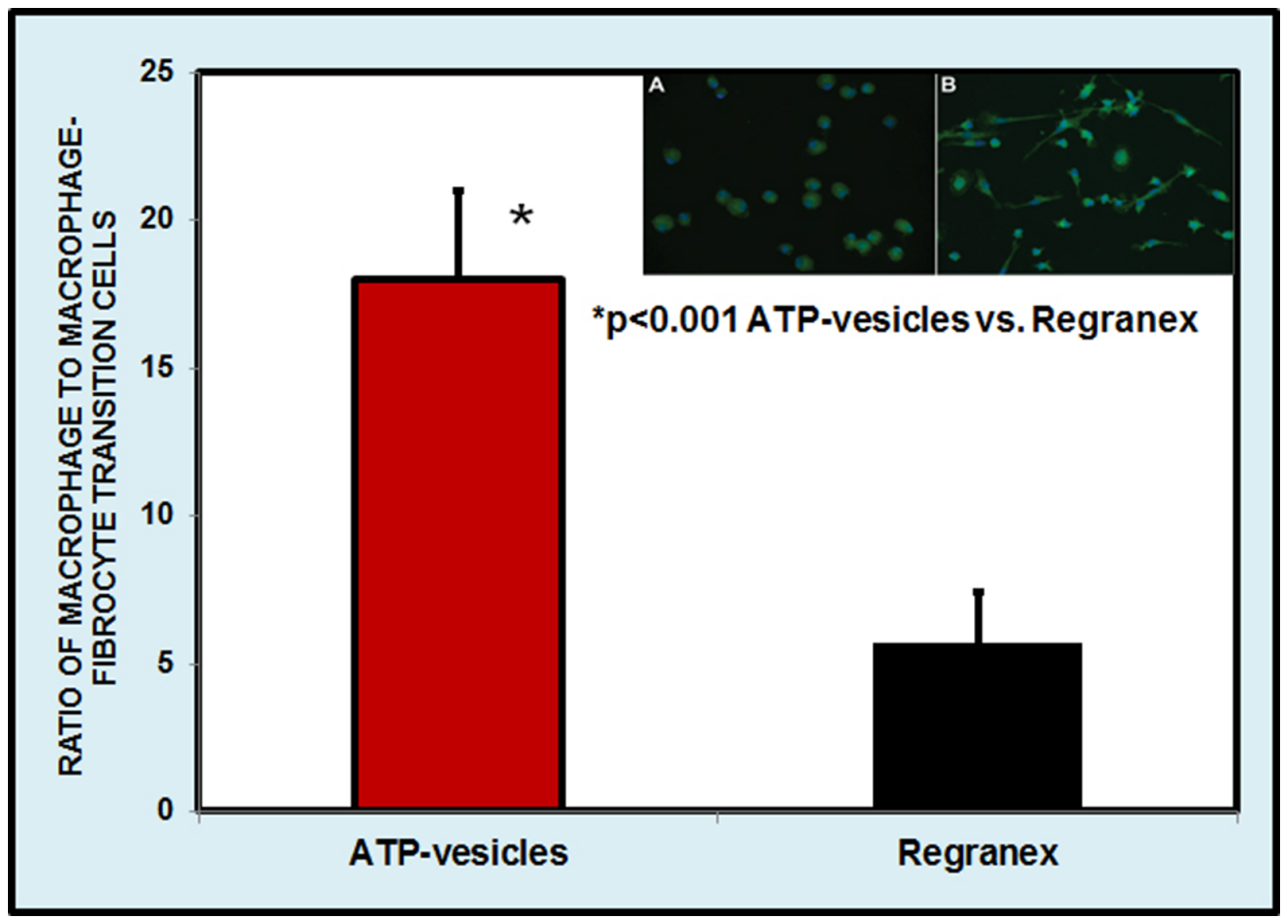
Macrophage culture at day 6. Most of the macrophages still maintain their original phenotype, whereas culturing with Regranex causes fibrocyte transformation. Inset shows the photos of the culture treated with ATP-vesicles (A) and treated with Regranex (B).

Wound macrophages are known to transform to fibrocytes, which produce more collagen. Interestingly, when ATP-vesicles were used in this study, this transformation was very much delayed. Figure 14 shows a comparison at day 6 of culture: Regranex caused fibrocyte transformation while most macrophages treated with ATP-vesicles retained their original phenotype.

### No Hypertrophic Scar Formation

**Figure 15:**
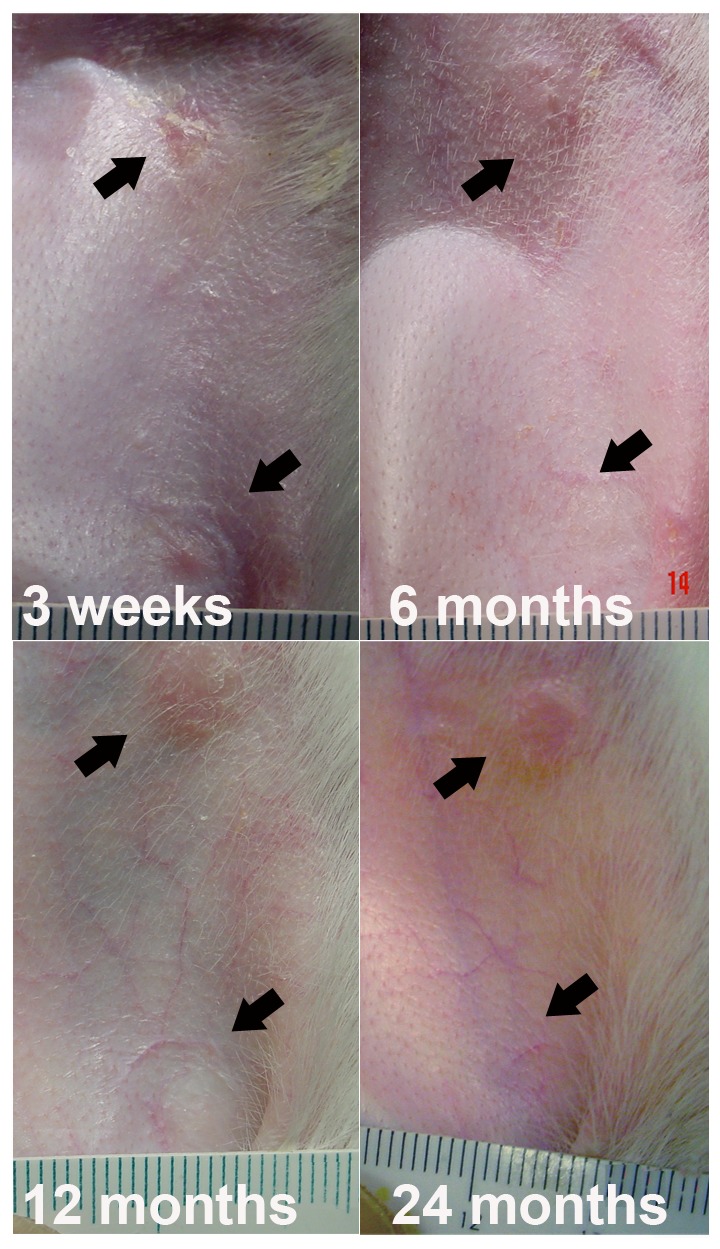
Appearance of healed wounds treated with ATP-vesicles. A representative sequential photomontage showing the smoothness of the healed wounds treated with ATP-vesicles even two years after treatment.

Rabbits are known for their tendency to produce hypertrophic scars [24], [25]. We followed some rabbits after their ATP-vesicle treated wounds were totally closed. No hypertrophic scar or any other unusual growth occurred in these wounds even after 2 years (Fig. 15).

## Discussion

Chronic wounds represent a silent epidemic that affects a large fraction of the world population and poses a major and gathering threat to public health and the economy [26]. The statement made in one of the first papers describing chronic wound treatment—that “Old ulcers in 1830 will be older ulcers in 1860”—unfortunately still applies today [27]. A search for ways to use the body's innate regenerative capacity to partially or completely repair tissue defects—naturally and without complex surgery—has been ongoing for more than a century. A surge in research in this area occurred in the 1930s and 1940s, but the end results were all disappointingly similar. In one elegant study reported in 1943, Howes tested the healing effects of various drugs (e.g., aquaphor, sulfadiazine, triethanolamine, Vaseline) on injured rabbit ears, and concluded that: 1) epithelialization begins after a latent period of 3–6 days, during which the underlying connective tissue is hardly regenerated at all; 2) a suitable granulating base is necessary for epithelialization to begin; and 3) the requirement for frequent dressing changes prolongs the latent period due to tearing away of the regenerating cells [28]. Scientific research since then has focused on the extracellular matrix, growth factors, and stem cells; however, no major breakthrough has occurred and Howes's archaic conclusions still remain valid to this day.

In the past few decades, thousands of dressings have been developed for wound care, and many showed effectiveness in animal experiments. However, in a recent systematic review by the International Working Group of the Diabetic Foot (IWGDF), with 1322 papers identified, the conclusion was drawn that little published evidence yet justifies the use of any of these newer therapies in human wounds [29]. Platelet-derived growth factors showed promise in various chronic wounds [30], [31], leading to Regranex (rhPDGF-BB, becaplermin) becoming the first FDA approved growth factor for topical application after an expensive phase III multicenter double-blind, placebo-controlled trial. Regranex was shown to be effective in the treatment of patients with diabetic neuropathic ulcers [6], [30] and was also used off-label to treat acute wounds, where it was reported to show almost 25% faster healing than an antibiotic ointment in randomized human volunteers [6].

In general, PDGF appears to be of central importance in the wound healing cascade, and is thought to stimulate cell migration, cell growth, and synthesis of other growth factors; thus, its natural function seems to involve the early phase of healing [32]. However, aggressive wound care is required to obtain good results and even so, the effectiveness of PDGF is not universally recognized [9], [33]. One possible problem involves the local protease-rich environment of a chronic wound, which has been shown to actively degrade and inactivate most growth factors. This finding may, in part, explain the altered levels of growth factors in chronic wounds, but it also suggests that topical therapeutic applications probably allow only a brief suboptimal exposure of the wound to the growth factor unless the protease-rich, pro-inflammatory wound environment is first addressed [34], [35].

In searching for better treatments for chronic wounds, we came up with the concept of intracellular energy delivery. This technique is based on the theory that more than 100 factors are involved in non-healing chronic wounds [36], [37], but deficient blood supply is one of the most important factors [38], [39]. Ischemia may not be the initiating factor for wounds such as diabetic foot ulcers because most of these ulcers start from a combination of neuropathy, pressure loading, and/or trauma. However, tissue ischemia is the main cause that hinders healing—wounds do not heal in tissue that does not bleed, whereas they always heal in tissue that bleeds extensively. Increasing the wound oxygen supply, such as through hyperbaric oxygen therapy, has not shown consistent results [40], [41] because a lack of oxygen is only one part of ischemic pathophysiology—the most critical consequence of ischemia is a decreased cellular energy supply [39], [42].

Energy is required in every aspect of the wound healing process: When a protein is built by linking its individual amino acids via peptide bonds, a significant input of chemical energy is required [43]. Other critical healing activities, such as cell migration and proliferation, leukocyte bactericidal function, membrane transport, signal transduction, and growth factor production all consume energy [44]. Small scale clinical trials of other growth factors such as bFGF have shown substantial effectiveness in non-ischemic situations, but their effects disappear in hypoxic dermal ulcer models [37], [45].

Efforts to deliver high-energy phosphates such as free Mg-ATP directly into the cytosol have not been successful because the bilayer cell membrane is impermeable to most water-soluble molecules, including Mg-ATP, which has no specific transport mechanisms [46]. In addition, the half-life of ATP in the blood is less than 40 seconds, making it impossible to be an effective treatment [47]. We have overcome these problems by encapsulating Mg-ATP in very small unilamellar lipid vesicles. The lipid vesicles have a composition similar to those of the cell membrane. When they come in contact with the cell membrane, they fuse with the membrane and deliver their contents into the cytosol [15], [19], [48].

During the initial vesicle development process, we compared the effectiveness of ATP vesicles with that of their individual ingredients alone (lipid vesicles only, free Mg-ATP only, and lipid vesicles plus Mg-ATP without encapsulation), culture media alone, or normal saline alone. In several test models (cell cultures, myocardial preservation, and wound healing) none of the control ingredients showed protective or healing effects comparable to ATP-vesicles [12], [13], [15], [19], [48]–[50]. The present study represents the first attempt to compare the effectiveness of Regranex with that of intracellular energy delivery therapy. Our results indicate that when ATP-vesicles are used, wound healing is enhanced compared to that seen with Regranex, and this enhancement is even more pronounced in ischemic wounds. However, the most unique finding is the extremely early and rapid generation of granulation tissue: Gross tissue generation was apparent in less than 24 hours after surgery. This tissue continued to grow and it had covered almost the whole cavity within 3–6 days. Similar growth was not seen in the control wounds treated with Regranex.

The early growth was composed mainly of macrophages, as shown by anti-Mac387 and further confirmed by CD68 and CD163 stains. These macrophages showed active proliferation, as indicated by PCNA and further confirmed by BrdU immunohistochemistry staining. The very early growth (within 1–2 days) appears somewhat pale, fragile, and edematous, without the traditional beefy look, so that it is easily mistaken for nonviable tissue. This growth quickly turned pink and solid looking after 2–3 days. When we tried to trim the overgrowth, it always had “roots” connected to the wound edge, and the “roots” changed from relatively weak at day 1 to stronger after 2–3 days. As time progressed, the cell numbers were reduced and were replaced by fibrous tissue [13]. However, reepithelialization started as early as 3 days postoperatively and was seen to tunnel through the granulation tissue until the top portion fell off, leaving behind perfectly healed wounds [13], [14].

This healing process appears to be totally different from the conventional process where fibrin, platelets, and red blood cells are the main components of the early provisional matrix, which is gradually replaced by granulation tissue after a lag of 3–6 days [51]. Regranex-treated wounds seem to follow the traditional healing pattern.

In medicine, an extremely rapid tissue growth is not always a desirable event unless it is well controlled. Neoplastic growth is a notable negative example. Sustained and unchecked growth (overgranulation) would also delay healing or could result in hypertrophic scar formation or keloids [25]. The growth in our ATP-vesicle-treated wounds is even faster than any tumorigenic growth. However, the healed wounds show no hypertrophic scar formation or any other growth, even two years after surgery. Like many other wound care specialists, when we first saw this phenomenon, we did not believe it because it appeared too good to be true. However, it has now been confirmed in more than 130 rabbits (over 1040 wounds), including diabetic wounds, as reported in past articles [13], [14]. Although rabbits have the ability to regenerate ear cartilage when the perichondrium is preserved, their skin regeneration capacity is essentially similar to that of other mammals [28], [52].

This unprecedented growth caused by ATP-vesicles may overcome the limitations of conventional wound healing processes and provide a new approach for tissue regeneration. Previously, the mechanisms underlying this unprecedented rapid growth were totally unclear to us, and the huge accumulation of macrophages in such a short period of time in relatively under-perfused acute wounds seemed unbelievable. However, a recent development has shed light on this unprecedented phenomenon, and now provides a very logical explanation.

Traditional textbook knowledge holds that progenitor cells and promonocytes proliferate and differentiate in the bone marrow. These cells enter the circulation to become monocytes, which are end cells. The monocytes migrate randomly from the peripheral blood into the tissues, where they become macrophages [53], [54]. However, a few sporadic previous reports [55], and especially a recent report in Science [56], challenged this old dogma by describing massive in situ macrophage proliferation during infection with the parasite *Litmosoides sigmodontis*, even when blood monocytes were depleted. More than 3 follow-up comments and reports by highly respected macrophage experts expressed high enthusiasm for this new finding [54], [57]–[59]. Our staining studies (PCNA plus BrdU antibody immunohistochemistry) have confirmed very active proliferation activity that reflects these previous findings.

What is special in our study is that no parasite or bacterial infection is involved. The mechanisms giving rise to the macrophage accumulation are not totally clear at this time, but must be different from those concerned with infection. The most likely pathway involves the ATP-dependent remodeling SWI/SNF complex, which uses the energy of ATP hydrolysis to rearrange chromatin structure—and in doing so, allows transcription of target genes to proceed [60]. The SWI/SNF complex comprises up to 12 components, which assemble into distinct complexes containing either BRG1 or Brm ATPase subunits. Both BRG1 and Brm are ubiquitously expressed in almost all tissues and they participate in cell proliferation. Indeed, more work is required to fully explore the mechanisms involved in ATP-vesicle driven wound healing. However, one issue is clear: since there is no blood supply at all to the wound cavity at the very earliest times, and since vascular regeneration always lags behind tissue growth, the exogenously provided intracellular Mg-ATP plays a key role in maintaining the survival and functioning of the cells in the wound cavity, and probably also in the wound wall. Without energy, these cells die quickly and become exudates requiring removal.

Huge macrophage accumulation in a wound cavity, even accompanied by other cells, does not make the growth a real granulation because the loose cell mass can die or dislodge easily. Our data show two more distinct downstream pathways that directly transform massive cell accumulation into granulation—direct collagen production and enhanced angiogenesis. These features were described in our previous reports [13]–[15]. In the present study, our aim was to explore the direct collagen production further by using macrophage culture and we were able to show that ATP-vesicles promoted macrophage collagen production and also, surprisingly, extended the life span of the macrophages.

The ability of macrophages to produce collagen is well established in other diseases [61]–[64]. The possibility of direct collagen production in wounds by macrophages was proposed two decades ago, but it is still largely dismissed by most wound care specialists [65], [66]. Collagen production by alternative activation is believed to occur via the arginine pathway that leads to proline production—a major component of collagen [67], [68]. The present results have provided evidence that massive accumulation of macrophages can be transformed directly into extracellular matrix through direct collagen production and neovascularization—completing the new healing process.

The comparison in this study was between Mg-ATP, which was encapsulated for intracellular delivery, and rh-PDGF-BB, which was not encapsulated. This fact does not invalidate this study. This is because most of growth factors function via activation of surface receptors. The receptor-bound growth factors are endocytosed and translocated to the cytosol and nucleus where they stimulated RNA and DNA synthesis, resulting in cell proliferation, differentiation, and tissue morphogenesis [69]–[72].

There are some limitations to this study. The scarcity of in depth study of in situ macrophage proliferation means that its pathways, especially those related to intracellular ATP delivery, are unclear at this time and require further study. The phenomenon of macrophage collagen production is seen in our study but this phenomenon is still controversial. More studies are also needed in this area.

Taken together our results show that: 1) full-thickness rabbit ear skin wounds, which have little or no skin contraction, are healed faster by ATP-vesicle treatment than by Regranex, and the effect is more pronounced in ischemic wounds; 2) intracellular ATP delivery produces very early and exuberant tissue regeneration; 3) the technique appears to cause in situ macrophage proliferation without the involvement of infection or inflammation; and 4) ATP-vesicle treatment enhances macrophage collagen production to further enhance wound healing without provisional matrix involvement. This new healing process and its related macrophage proliferation may advance our basic understanding of macrophage biology and will have clinical implications in wound care and other related diseases.
